# Measuring change in adolescent physical activity: Responsiveness of a single item

**DOI:** 10.1371/journal.pone.0268459

**Published:** 2022-06-03

**Authors:** Paul O’Halloran, Courtney Sullivan, Kiera Staley, Matthew Nicholson, Erica Randle, Adrian Bauman, Alex Donaldson, Nicola McNeil, Arthur Stukas, Annemarie Wright, Michael Kingsley

**Affiliations:** 1 School of Psychology and Public Health, La Trobe University, Bundoora, Australia; 2 Centre for Sport and Social Impact, La Trobe University, Bundoora, Australia; 3 Holsworth Research Initiative, La Trobe University, Bendigo, Australia; 4 Monash University, Malaysia, Subang Jaya, Malaysia; 5 School of Public Health, Sydney University, Sydney, Australia; 6 La Trobe Business School, La Trobe University, Bundoora, Australia; 7 The University of Melbourne (Honorary), Parkville, Australia; 8 Department of Exercise Sciences, University of Auckland, Auckland, New Zealand; University of Pavia: Universita degli Studi di Pavia, ITALY

## Abstract

Self-report measures are frequently used to assess change in physical activity (PA) levels. Given the limited data from adolescent populations, the primary objective of this study was to examine the responsiveness of a single item measure (SIM) of PA for adolescents to detect change in moderate-to-vigorous physical activity (MVPA) using accelerometer data as the reference measure. A secondary objective was to provide further data on the validity of the measure at one point in time. The validity of the SIM to determine the number of days ≥60 minutes of MVPA was based on data from 200 participants (62% female; age: 14.0 ± 1.6 years) and analysis of change was based on data from 177 participants (65% female; age: 14.0 ± 1.6 years). Validity of change in days ≥60 minutes of MVPA was examined through agreement in classification of change between the SIM and accelerometry as the reference measurement and Spearman’s correlation. Cohen’s d and standardised response means were used to assess the responsiveness to change of the measure. The responsiveness of the SIM and accelerometer data were comparable and modest (0.27–0.38). The correlation for change in number of days ≥60 minutes MVPA between the SIM and accelerometery was low (r = 0.11) and the accuracy of the SIM for detecting change, using accelerometry as the reference, was only marginally above chance (53%). Therefore, the adolescent version of the SIM is adequate for assessing PA at a single time point but not recommended for assessing change.

## Background

The measurement of physical activity (PA) is important for identifying the prevalence and determinants of PA and for evaluating the effectiveness of interventions designed to change activity levels [1]. It is important to accurately measure PA for children and adolescents given the beneficial effects of PA on numerous health outcomes including bone, cardio-metabolic and mental health, and cardiorespiratory and muscular fitness [2–5]. There has been an increased use of device-based measurement of PA (e.g. accelerometers), largely due to the demonstrated reliability and validity of these instruments [1, 6]. However, there are limitations associated with using accelerometers with children and adolescents, including cost, feasibility and poor compliance [7–10]. Accordingly, self-report measures remain popular for assessing PA in adult, child and adolescent populations given that they can be easily administered to large representative samples at low cost [11–13].

Self-report PA questionnaires comprise different PA domains, intensities and categories so it is challenging to determine the most appropriate instrument. A previous systematic review of adolescent PA self-report measures concluded that, of the 76 questionnaires examined, the only questionnaire with acceptable construct validity in assessing adolescent PA in both males and females was the Greek version of the 3-Day Physical Activity Record (3DPARecord) [14]. However, using detailed questionnaires like the 3DPARecord pose a greater respondent burden and higher cognitive demands when compared to shorter questionnaires [15]. Consequently, single item measures (SIM) of PA have been developed for various populations [16–18]. A SIM was developed and validated by Milton and colleagues to assess PA in adults. It has one question asking participants to report the number of days they undertook at least 30 minutes of moderate to vigorous physical activity (MVPA) in the last week related to leisure or transport [19]. An adolescent SIM has also been developed, which asks participants to report the number of days they undertook at least 60 minutes of MVPA in the last week related to leisure or transport [20]. The criterion validity and test-retest reliability of the adult SIM has been supported based on comparisons with accelerometer data [19, 21–23]. The one study that examined the validity of the adolescent SIM [20] reported acceptable concurrent validity and test-retest reliability for the adolescent SIM in assessing PA in adolescents when compared to accelerometery.

Self-report measures are often used in intervention studies to assess changes in PA levels. Therefore, it is important that they provide a valid assessment of change [24–26]. In recent times, the validity of self-report measures to detect changes in MVPA has been assessed using accelerometer data as the reference measure [24, 27–32]. Some studies report acceptable agreement between self-report measures and accelerometry derived changes in PA [24, 27, 28], while others demonstrate limited validity [24, 29, 31, 32]. Additionally, these studies were specific to adult populations; no investigation to date has examined the responsiveness of self-report measures to detect MVPA change in adolescents using accelerometery as the reference measure.

More recently, investigations examining the responsiveness of self-report measures to detect temporal changes in PA using accelerometer data have utilised a study design whereby increases in PA were actively promoted [22, 28, 29, 31, 32]. Of note, recent studies assessing the responsiveness of measurement tools to assess changes in PA using effect size statistics such as Cohen’s d and standardised response mean have generally reported moderate responsiveness and comparability between measurement tools [22, 28]. For example, the only investigation to date examining the responsiveness of a SIM to detect change in PA in adults using accelerometer data as the reference measurement reported moderate to high responsiveness for both measures (SRM all ≥0.57 [22]. The authors concluded that the responsiveness of the SIM and accelerometer to detect changes in PA in adults was comparable between measurement tools.

Although there is an extensive literature assessing the validity of self-report measures of PA in adolescents against accelerometry at a single time point [14, 20, 33, 34], the evaluation of responsiveness to change in PA has only been reported in adult populations. Data about the validity of a SIM for adolescents is limited and no previous study has examined the responsiveness of the measure to detect change in MVPA in an adolescent population using accelerometer data as a reference measurement. Therefore, the primary aim of this study was to assess the responsiveness of an adolescent version of the SIM to detect change in MVPA in an adolescent population, using accelerometer data as a reference measurement, when change in MVPA was promoted through an intervention. A secondary aim was to obtain further validity data for this adolescent version of the SIM compared to accelerometer data for assessing the number of days ≥60 minutes MVPA at one point in time.

## Methods

### Participants

Participants were recruited via their parents through an electronic newsletter distributed using a restricted email list to staff at a regional campus of an Australian university. Participants were eligible if they were between 12 and 17 years of age and passed screening with the Physical Activity Readiness scale [35]. Informed written assent and consent were provided by the participant and their parent/guardian if the participant was under 16 years of age. Two hundred and seven participants provided informed consent and the La Trobe University Human Ethics Committee approved the study (HEC18301).

### Procedure

All participants, accompanied by a parent/guardian, attended four face-to-face sessions during the study to instruct them about the program, fit the accelerometer and complete a questionnaire (including the SIM and demographic questions). The first session was an information session during which participants completed the questionnaire and received a triaxial accelerometer (GT3X+; Actigraph LLC, USA) which was placed above their right hip on a belt. Accelerometers were set to record triaxial accelerations at 100 Hz and calibrated according to the manufacturer’s guidelines. Participants were asked to wear the accelerometer during all waking hours, except when bathing or in water, for two 7-day data collection periods (Week one and Week two).

At the first data collection period (Week one), participants were asked to maintain usual routines. Participants were sent daily text message reminders to encourage accelerometer wear. After completing this 7-day period, participants attended the second face-to-face session to return the accelerometer and complete the SIM, which asks participants: *In the past week*, *on how many days have you done a total of 60 minutes or more of physical activity*, *which was enough to raise your breathing rate*? *This may include sport*, *exercise*, *and brisk walking or cycling for recreation or to get to and from places*. Participants then completed a week without monitoring. They then attended the third face-to-face session where they were fitted with the accelerometer for the second 7-day data collection period (Week two).

During the second data collection period (Week two), the intention was to increase PA through the introduction of several intervention components. Participants were encouraged through an activity challenge to increase their running and/or walking during this period and daily text message reminders were sent with tips for increasing activity as well as encouraging accelerometer wear. Participants were sent daily text messages with ideas for how to increase steps, such as “go for a walk around the school’s oval”. They were also offered gift cards for completing the activity challenge with additional prizes awarded to individuals and teams (if that was the preferred option) who increased their accelerometry-derived step count between assessments by at least 25%. After completing this 7-day period, participants attended the fourth session to return the accelerometer and complete the SIM for the final time.

### Data analysis

Accelerometer signals were downloaded and analysed using the manufacturer’s software (Actilife version 7.0; Actilife Corp., USA). The vector magnitude, accumulated in 1-minute epochs, was used to determine non-wear time using the Choi wear time algorithm [36]. Accelerometry wear time minimums for inclusion in subsequent data analyses were an average of 8 hours per day with wear time recorded on all 7 days of the week. Sensitivity analyses were performed to evaluate the influence of wear time on results by including only data for participants who had wear times of ≥8 hours on all 7 days of both weeks. Valid wear time data were classified as being MVPA using the Evenson Children algorithm, where >2296 counts per minute were classified as MVPA [37]. The total time spent in MVPA was accumulated for each day. The accelerometry criterion of the number of days meeting ≥60 minutes of MVPA during Week one and Week two was determined for all participants.

### Statistical analysis

IBM SPSS Statistics for Windows, version 25.0 (IBM Corp., Armonk, N.Y., USA) was used for statistical analyses. Differences in the distributions of number of days ≥60 minutes MVPA between females and males were assessed using Pearson’s chi-squared test. The assumption of normality for the unit of measurement in this study (days of ≥60 minutes MVPA) was assessed by observation of the distribution and confirmed by the Shapiro-Wilk test. Days of ≥60 minutes MVPA were not normally distributed, therefore group data are presented as medians and interquartile range (IQR). Given the lack of normality, relationships between SIM and accelerometer derived MVPA were assessed using Spearman’s rank correlations. Relative and absolute agreement between SIM and accelerometer derived MVPA were examined separately. Relative agreement was assessed using K statistics and percent agreement. Absolute agreement between SIM and accelerometer derived MVPA was evaluated by plotting the difference in days (SIM minus accelerometer derived MVPA) against median days of ≥60 minutes MVPA from SIM and accelerometer [22]. As the distribution of the residuals did not show significant heteroscedasticity, median and IQR for these ordinal distributions were calculated and are presented as a figure.

The responsiveness and validity of the adolescent SIM to detect change in MVPA were compared to accelerometer data as the reference measurement. Estimates for the response to change (effect sizes) for this SIM and accelerometer data were calculated using Cohen’s d and the standardised response mean (SRM). Cohen’s d was calculated by taking the mean of individual change scores divided by the pooled standard deviation of the scores at both time points. SRM was calculated as the mean change in the number of days ≥60 minutes MVPA (i.e., change from baseline to follow up) divided by the standard deviation of individual change scores. Several additional measurements of agreement were used to assess the validity of this SIM to detect change, using accelerometry as the reference measurement. Effect size of the intervention measured by both the adolescent SIM and accelerometer were qualitatively interpreted using the following criteria: <0.20 = trivial, 0.20 to <0.50 = small, 0.50 to <0.80 = moderate, and >0.80 = large [38]. Inter-measurement reliability and association between the change in the number of days ≥60 minutes MVPA (Week two–Week one) as recorded by the adolescent SIM and accelerometry were evaluated by k statistic and Spearman’s rank correlations, respectively. In addition, agreement was evaluated between this SIM and accelerometry derived MVPA to classify change in PA as being “increased”, “decreased” or “no change”.

## Results

Of the 207 adolescents recruited to this study, seven participants (3%) were excluded from analyses because they did not attend the first face-to-face session (n = 6) or did not meet the accelerometer wear time requirements for either week (n = 1). Characteristics of the included participants are presented in Table 1. Valid data were available for 200 participants (125 females; age: 14.0 ± 1.6 years; body mass index: 21.9 ± 4.1 kg/m^2^) for at least one week of data collection. Valid data were available for both weeks for 177 participants (116 females; age: 14.0 ± 1.6 years, body mass index: 21.9 ± 4.1 kg/m^2^) and these data were included in analyses of change in PA from Week one to Week two. For sensitivity analyses, 76 participants had wear times of ≥8 hours per day for all 7 days of both weeks (54 females; age: 13.8 ± 1.5 years; body mass index: 21.3 ± 3.9 kg/m^2^).

**Table 1:** Characteristics of participants included for both phases of the analyses.

	Valid data for one week (n = 200)			Valid data for both weeks (n = 177)		
Characteristics	Male *n* (%)	Female *n* (%)	Total *n* (%)	Male *n* (%)	Female *n* (%)	Total *n* (%)
Place of birth						
Australia	71 (97)	115 (92)	188 (94)	58 (97)	106 (92)	166 (94)
Europe	1 (1)	6 (5)	7 (4)	1 (2)	6 (5)	7 (4)
Other	1 (1)	3 (2)	4 (2)	1 (2)	2 (2)	3 (2)
Both parents born in Australia	66 (90)	97 (78)	165 (83)	54 (90)	90 (78)	146 (83)
English spoken at home	72 (99)	119 (95)	193 (97)	59 (98)	110 (96)	171 (97)
Aboriginal/Torres Strait Islander	1 (1)	1 (1)	2 (1)	1 (2)	1 (1)	2 (1)
	Mean ± SD	Mean ± SD	Mean ± SD	Mean ± SD	Mean ± SD	Mean ± SD
Age (years)	14.1 ± 1.8	14.0 ± 1.5	14.0 ± 1.6	14.1 ± 1.8	13.9 ± 1.5	14.0 ± 1.6
Stature (cm)	166 ± 12	162 ± 7	164 ± 9	166 ± 12	162 ± 6	163 ± 9
Body mass (kg)	60.2 ± 15.9	57.9 ± 12.0	58.9 ± 13.5	60.0 ± 15.8	57.9 ± 12.0	58.7 ± 13.4

Valid = wear time average ≥ 8 hours per day for 7 days of the week. Total includes 2 participants who identified as gender diverse or stated “prefer not to say”. m = mean; SD = standard deviation.

### Agreement between the adolescent SIM and accelerometry at a single time point

Frequency data of days with ≥60 minutes of MVPA for Week one and Week two are presented in Table 2. The number of adolescents achieving seven days ≥60 minutes MVPA via the self-report and accelerometry in Week one was 13 (7%) and 2 (1%), respectively. In Week two, the number of adolescents achieving seven days ≥60 minutes MVPA via the self-report and accelerometry was 26 (14%) and 7 (4%), respectively. Sensitivity analyses, using participants who achieved ≥8 hours per day on all 7 days of both weeks, revealed that the proportion of adolescents who achieved seven days ≥60 min for Week one and Week two were 8% and 13% (self-report), and 3% and 7% (accelerometry).

**Table 2:** Frequency of days with ≥60 minutes of moderate-to-vigorous intensity physical activity (MVPA) at a single time point as measured using accelerometry and single item measure (SIM) self-report.

	Week one		Week two	
Days of ≥60 min MVPA	Accelerometer MVPA	self-report SIM	Accelerometer MVPA	self-report SIM
	n (%)	n (%)	n (%)	n (%)
0	31 (16%)	5 (3%)	20 (11%)	2 (1%)	
1	39 (20%)	17 (9%)	28 (15%)	5 (3%)	
2	38 (20%)	37 (19%)	30 (16%)	20 (11%)	
3	22 (11%)	29 (15%)	31 (17%)	40 (22%)	
4	27 (14%)	41 (21%)	30 (16%)	30 (16%)	
5	22 (11%)	34 (18%)	26 (14%)	36 (20%)	
6	11 (6%)	16 (8%)	10 (5%)	23 (13%)	
7	2 (1%)	13 (7%)	7 (4%)	26 (14%)	
**Median (IQR)**					
All participants	2 (1–4)	4 (2–5)	3 (1–4)	4 (3–6)	
Female	2 (1–4)	3 (2–5)	2 (1–4)	4 (3–5)	
Male	3 (2–5)	4 (3–6)	4 (3–5)	5 (3–6)	

**Fig 1:**
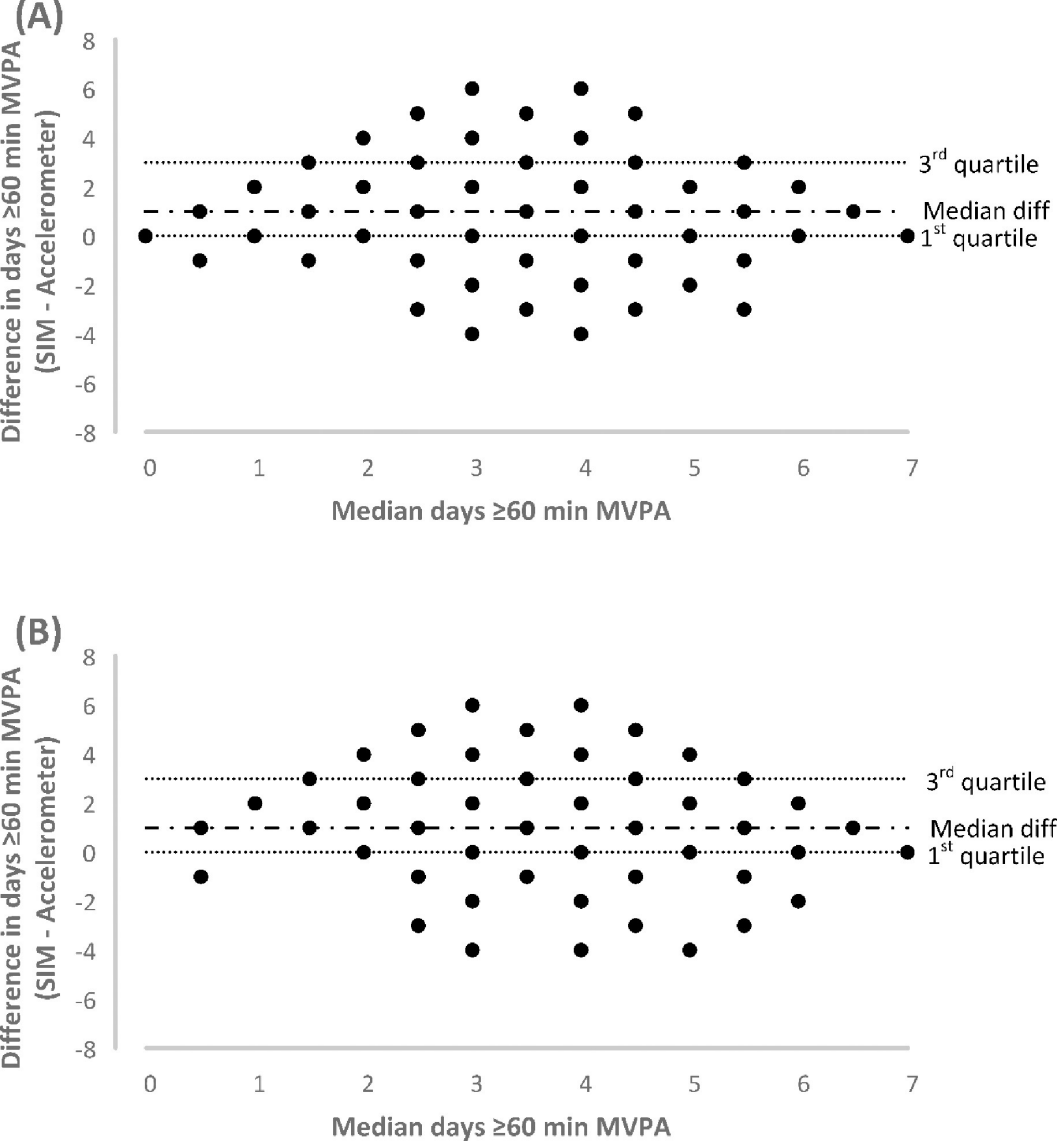
Difference in days ≥60 minutes of moderate-to-vigorous intensity physical activity (MVPA) between the single item measure (SIM) and accelerometry: (A) Week one and (B) Week two. Median difference and inter-quartile range for the ordinal distributions are presented as dotted horizontal lines against the median of the two measures (single-item and accelerometry).

Correlations for the number of days ≥60 minutes MVPA between the SIM and accelerometry were r = 0.35 (p < 0.001) and r = 0.30 (p < 0.001) for Week one and Week two, respectively. Comparable correlations were evident in the sensitivity analyses for Week one (r = 0.38) and Week two (r = 0.25). The median differences between the number of days with ≥60 minutes of MVPA between the SIM and accelerometry in both Week one and Week two were 1 day (IQR: 0 to 3 days) (Fig 1). Sensitivity analyses produced similar results for Week one (median: 1 day, IQR: 0 to 2 days) and Week two (median: 1 day, IQR: 0 to 3 days).

Although distributions for number of days that adolescents achieved ≥60 minutes of MVPA via accelerometry were different for females when compared to males for week 1 (*X*^*2*^_(7)_ = 15.92, p = 0.026; Table 2), these were not different in week 2 (*X*^*2*^_(7)_ = 8.62, p = 0.281; Table 2). Agreement between self-report and accelerometry at a single time point was comparable for females and males (supplementary data: Doi.org/10.26181/19426238.v1).

### Ability of the adolescent SIM to detect change in physical activity

Almost half (49%) of participants increased the number of days they achieved ≥60 minutes MVPA, while approximately one quarter (27%) did not change and one quarter (25%) decreased the number of days they achieved ≥60 minutes MVPA (Table 3). Sensitivity analyses, including only participants who achieved ≥8 hours per day for all 7 days of both weeks, for the proportion of participants who increased, did not change and decreased the number of days they achieved ≥60 minutes MVPA were 41%, 30% and 28%, respectively.

**Table 3:** Binary classification tests for the single item measure versus accelerometer for detecting increase, decrease and no change in number of days ≥60 minutes MVPA from Week one to Week two.

	Increase in number of days	No change in number of days	Decrease in number of days
Participants in category (n, %)	85 (49%)	47 (27%)	43 (25%)
Accuracy (%)	54.3 (46.6 to 61.8)	62.3 (54.7 to 69.5)	66.9 (59.4 to 73.8)
Sensitivity (%)	52.9 (41.8 to 63.9)	36.2 (22.7 to 51.5)	25.6 (13.5 to 41.2)
Specificity (%)	55.6 (44.7 to 66.0)	71.9 (63.3 to 79.5)	80.3 (72.5 to 86.7)
Positive predictive value (%)	52.9 (45.3 to 60.4)	32.1 (22.8 to 43.0)	29.7 (18.6 to 43.9)
Negative predictive value (%)	55.6 (48.3 to 62.6)	75.4 (70.7 to 79.6)	76.8 (73.2 to 80.1)

The SIM correctly detected 54% of participants who increased their MVPA (sensitivity). The proportion of participants who reported an increase in PA and did according to accelerometry (Positive predictive value) was 53% for MVPA. The SIM correctly detected 56% of people who did not increase (i.e., no change or decreased) their MVPA (specificity). The negative predictive values demonstrate that the SIM correctly classified approximately 56% who did not increase their PA according to accelerometry. Sensitivity analyses for those individuals who increased MVPA demonstrated that accuracy (55%), sensitivity (47%), specificity (63%), positive predictive value (56%) and negative predictive value (55%) were consistent with data in Table 3. Additionally, results for the sensitivity analyses were comparable to those presented in Table 3 for those participants who did not change or decreased their MVPA (Supplementary data: doi.org/10.26181/19426238.v1).

Agreement between accelerometry and SIM at a single time point was comparable for females and males (supplementary data: doi.org/10.26181/19426238.v1).

Accuracy, sensitivity, specificity, positive predictive value and negative predictive value are presented as point estimates and 95% confidence intervals. Median changes in the number of days ≥60 minutes.

Change in the number of days that participants achieved ≥60 minutes MVPA from Week one to Week two using the self-report and accelerometry for MVPA were both 0 days (IQR: 0 to 2), which was consistent with results from the sensitivity analyses. The correlation for change in number of days ≥60 minutes MVPA between the SIM and accelerometery was r = 0.11 (p = 0.160), which was consistent with results from the sensitivity analyses (r = 0.10).

Responsiveness was moderate for the SIM (0.38, 95% CI: 0.23 to 0.53) and accelerometry (0.30 95% CI: 0.17–0.45). Cohen’s d values for change based on the SIM and accelerometry were 0.37 (95% CI: 0.23–0.53) and 0.27 (95% CI: 0.14–0.40), respectively. Inter-measurement reliability for change in PA classification between the SIM and accelerometry was negligible with a confidence interval crossing zero (k = 0.02, 95% CI: -0.05 to 0.09). Corresponding inter-measurement reliability for change in the sensitivity analyses was also negligible (k = 0.10, 95% CI: 0.03 to 0.17).

## Discussion

This is the first study to assess the responsiveness of a SIM to detect change in days of MVPA against accelerometer data using an adolescent population. Our main finding was that the adolescent SIM and accelerometer data displayed similar responsiveness in evaluating change in the number of days that adolescents perform ≥60 minutes of MVPA. However, the validity of this SIM to detect accelerometer-defined change in PA was poor. The results suggest that the adolescent version of the SIM is more useful to assess PA at a single time point than to assess temporal changes in PA within interventions for adolescents.

### Agreement between SIM for adolescents and accelerometry at a single time point

Comparable to previous research in adolescents [20], the adolescent version of the SIM was moderately correlated with the number of days of ≥60 minutes MVPA recorded by accelerometry in both Week one (r = 0.35) and Week two (r = 0.30). While there is no consensus within the literature on an acceptable correlation coefficient for youth when assessing the validity of PA measured via self-report questionnaires, a systematic review reported that validity correlation coefficients for self-report measures for this population typically range between 0.30 and 0.40 [39]. Therefore, the moderate correlation between the adolescent SIM and accelerometer data reported in the present study is typical of findings from studies using longer self-report PA measures including the Previous Day Physical Activity Recall (PDPAR) and the Multimedia Activity Recall for Children and Adolescents (MARCA) [39]. The adolescent version of the SIM demonstrates similar validity in assessing PA at a single time point when compared with longer currently available adolescent PA questionnaires.

Absolute agreement between the adolescent version of the SIM and accelerometer data indicates that participants overestimated the number of days ≥60 minutes of MVPA on the self-report measure by an average of 1 day. This appears to be typical of previous research investigating the agreement between self-report measures and accelerometer data where most, but not all [33], reported that self-report measures overestimate minutes of PA performed in both adolescents [33, 40] and adults [41]. Of note, these studies assessed the time spent in different intensity categories of PA as opposed to the number of days where a threshold was met, such as the unit of measurement used in the present study. In studies that have assessed the absolute agreement between the adult version of the SIM and accelerometer data in adults, the SIM underestimated the number of days ≥30 minutes of MVPA in an adult population [21, 22] and is therefore in contrast to the present findings.

### Ability of the SIM to detect change in physical activity

The intervention in the current study resulted in 49% of participants increasing days of >60 minutes of MVPA by one or more days from Week one to Week two. Responsiveness of the adolescent version of the SIM and accelerometer data were comparable and modest (0.27–0.38). This is in line with O’Halloran and colleagues [22] who reported moderate and comparable responsiveness to change in PA between the SIM and accelerometer data in 90 adult participants (79% female, 47 ± 11 years).

This study found a weak correlation (r = 0.11) between change in the number of days ≥60 minutes MVPA detected by the SIM and accelerometer. This study is the first to report on the validity of a self-report measure to detect change in the number of days ≥60 minutes MVPA in an adolescent population. When compared to a study investigating the responsiveness and validity of a SIM to detect change with accelerometer data as the reference measurement in an adult population [22], the correlation in this study is lower (r = 0.11 versus r = 0.36). Similarly, the relative agreement between the SIM and MVPA reported in the present study is lower than that reported by previous research using adult participants and other self-report measures like the short REGICOR questionnaire [30], AAS [32] and the GPAQ [27]. Although the lack of relative agreement between the adolescent version of the SIM and MVPA suggests that this SIM might not be suitable for detecting changes in PA in adolescent populations, this study is the first to investigate the validity of change in PA using the SIM in adolescents, and so further research is required to confirm this finding.

Inter-measurement reliability between the SIM for adolescents and accelerometer for change in MVPA from Week one to Week two was low (k = 0.02). Furthermore, the confidence intervals crossed zero so no agreement may exist between measurement methods. This finding demonstrates a lack of relative agreement between the SIM and accelerometer in detecting change in the number of days ≥60 minutes MVPA in an adolescent population.

The overall accuracy of the adolescent version of the SIM to detect increases in PA was 54%, where the unit of change is days ≥60 minutes MVPA, with the sensitivity only marginally better than chance. The proportion of adolescents who self-reported increasing PA with this SIM and also increased their PA according to accelerometry (positive predictive value) was also marginally better than chance (53%). The sensitivity and positive predictive value of the adolescent SIM for detecting change are considerably lower than those reported for the adult SIM [22].

The interpretation of the findings from this study should be considered in the light of several limitations. Although the sample was unbalanced with 64% of the sample being female, and on average the females were less active than males, agreement between the self-report and accelerometry to detect days of sufficient PA as well as change in PA were similar for adolescent females and males in this study. Furthermore, the sample selection process was not representative. Thus, further research is needed to confirm these findings using different population groups. The use of accelerometer data as the reference measurement for comparison with the self-report measure was another potential limitation. As highlighted by in previous research [9, 14, 20], using accelerometers as a gold standard of PA collection is contentious given the variation in PA estimates, varying choice of cut-points for intensity levels and definitions of valid days and wear time [8, 42, 43]. Thus, limitations associated with both accelerometry and self-report data need to be considered when interpreting differences in physical activity between the two tools. Moreover, accelerometers can underestimate PA if they are not consistently worn in some settings such as swimming and cycling [44, 45]. Despite these limitations using accelerometers to collect PA data, sensitivity analyses confirmed consistency of results when strict wear time requirements were applied, and the validation of self-report measures against accelerometer data remains standard practice. This is largely due to their practicality when compared with other PA and energy expenditure measurement methods such as doubly labelled water [46, 47].

## Conclusion

Correlations between the adolescent SIM and accelerometer derived MVPA were moderate when days ≥60 minutes of MVPA were assessed at a single time point. When compared to accelerometry, the adolescent SIM tended to over-estimate the number of days ≥60 minutes of MVPA in the present sample. When evaluating the change in the number of days ≥60 minutes of MVPA performed, the adolescent SIM and accelerometer displayed similar responsiveness; however, the validity of this SIM to detect change in PA using multiple indicators was poor. This illustrates the value of analysing multiple indicators of agreement in change when comparing the adolescent version of the SIM to accelerometry as the reference measurement. These data suggest the adolescent SIM is as useful as other self-report measures in assessing the number of days ≥60 minutes of MVPA. However, in contrast to the adult SIM, the self-report measure is not recommended for detecting change in PA in an adolescent population.

## Supporting information

S1 File (DOCX)

S2 File (DOCX)
