# Supplementary material for: Measuring change in adolescent physical activity: Responsiveness of a single item
Source: PLoS One. 2022 Jun 3;17(6):e0268459. doi: 10.1371/journal.pone.0268459 (PMC9165893; doi:10.1371/journal.pone.0268459)
Supplement: S1 File — (DOCX) [file pone.0268459.s001.docx]

Our data are publicly available via the OPAL Repository. Direct links to these files are provided throughout the manuscript as DOIs. ([doi.org/10.26181/19426238.v1](https://doi.org/10.26181/19426238.v1))
