# Supplementary material for: Measuring change in adolescent physical activity: Responsiveness of a single item
Source: PLoS One. 2022 Jun 3;17(6):e0268459. doi: 10.1371/journal.pone.0268459 (PMC9165893; doi:10.1371/journal.pone.0268459)
Supplement: S2 File — (DOCX) [file pone.0268459.s002.docx]

Financial Disclosure:
This research was supported by the Victorian Health Promotion Foundation (VicHealth), [www.vichealth.vic.gov.au](https://protect-au.mimecast.com/s/mKxWCnxyDrCk4VG1cPt9r7?domain=vichealth.vic.gov.au). One of the co-authors was a staff member of the funding body at the time of data collection, and as such the funder had a role in the preparation of the manuscript. However, the funder had no role in the design of the study or the analysis of data.
